# Oestrogen receptor testing and initiation of adjuvant endocrine therapy in women with ductal carcinoma in situ: a population-based study

**DOI:** 10.1007/s10549-026-08072-7

**Published:** 2026-09-02

**Authors:** Qian Chen, Ian Campbell, Mark Elwood, Alana Cavadino, Phyu Sin Aye, Sandar Tin Tin

**Affiliations:** 1 Department of Epidemiology and Biostatistics, Faculty of Medical and Health Sciences, University of Auckland, Auckland, New Zealand; 2 Department of Surgery, Faculty of Medical and Health Sciences, University of Auckland, Auckland, New Zealand; 3 Department of Pharmacology, Faculty of Medical and Health Sciences, University of Auckland, Auckland, New Zealand; 4 Division of Health, University of Waikato, Hamilton, New Zealand; 5 Cancer Epidemiology Unit, Oxford Population Health, University of Oxford, Oxford, UK

**Keywords:** Ductal carcinoma in situ, Oestrogen receptor testing, Adjuvant endocrine therapy, Regional variation

## Abstract

**Introduction:**

Use of adjuvant endocrine therapy (ET) for ductal carcinoma in situ (DCIS) varies widely in clinical practice. We investigated oestrogen receptor (ER) testing, the initiation of ET, and the effect of ET on outcomes for DCIS in New Zealand.

**Methods:**

Women with DCIS (2000–2022) were identified from the New Zealand Breast Cancer Foundation National Register and linked to the national pharmaceutical data. Logistic regression identified factors associated with ER testing, and cumulative incidence of breast cancer events was estimated with death as a competing risk.

**Results:**

Among 5813 women with DCIS, 894 patients (15.4%) had ER testing, with the rate increasing from 11.1% in 2000 to 23.3% in 2006, before declining to 13.9% in 2022. The Waikato region contributed 65.6% of ER tests. In Waikato, testing was more likely among women who received adjuvant radiotherapy after breast-conserving surgery, likely reflecting a site-specific clinical practice. In other regions, testing was more frequent among older women, Māori ethnicity, those with symptomatic presentation, and those with DCIS size > 20 mm, potentially reflecting selective testing. Among 729 patients with ER-positive DCIS, only 183 (25.1%) commenced ET, of whom 80.3% were in Waikato. ET users had the lowest cumulative risk of breast cancer event, but differences were not statistically significant.

**Conclusions:**

ER testing and initiation of ET for DCIS remained low, except in Waikato, where clinical centres participated in ET trials and ER testing became more common. Standardised ER testing and greater clinician participation in clinical trials may lead to improved evidence-based care.

**Clinical trial number:**

Not applicable.

**Supplementary Information:**

The online version contains supplementary material available at https://doi.org/10.1007/s10549-026-08072-7.

## Introduction

Ductal carcinoma in situ (DCIS) accounts for about one-fourth of all breast cancer cases (invasive and in situ) identified through screening [1, 2]. The standard treatment for DCIS involves mastectomy or breast-conserving surgery (BCS), with or without adjuvant radiotherapy (RT), and/or endocrine therapy (ET), to reduce the risk of recurrence.

Previous population-based studies have found that approximately 65–80% of DCIS cases are oestrogen receptor positive (ER+) [3–5]. Meta-analyses of clinical trials and observational cohort studies have shown that ET reduces the risk of recurrence in DCIS; however, no survival benefit has been established [6]. Since 2020, the American Society of Clinical Oncology/College of American Pathologists (ASCO/CAP) and National Comprehensive Cancer Network (NCCN) guidelines have recommended ER testing for patients with DCIS to help guide decisions on the use of ET, while progesterone receptor (PR) testing is not required [7, 8]. Yet, the use of ET for ER+ DCIS, such as tamoxifen (TAM) and aromatase inhibitors (AIs), has varied widely in clinical practice, ranging from 16% to 71% [4, 9–13].

According to the 2009 New Zealand Early Breast Cancer Treatment Guidelines [14], ET is recommended for women with ER+ invasive breast cancer, while its use in ER+ DCIS should be guided by individual circumstances. Previous studies found that approximately 85% of invasive breast cancers in New Zealand were ER+, and nearly all of these patients received ET [15]. Data from the Breast Surgeons of Australia and New Zealand Quality Audit show that among women with DCIS who underwent surgery, only approximately 25% were ER + and 68% had unknown ER status [16]. Centres participating in clinical trials may have greater adherence to evidence-based practice [17]. The Waikato region, with its well-established breast cancer research infrastructure, has been a leading region for participation in breast cancer clinical trials in New Zealand. Given that the evidence on ER testing and the use of ET for DCIS in New Zealand remains limited, this population-based study investigated trends of ER testing, subsequent use of ET and breast cancer outcomes in women with DCIS.

## Methods

### Data sources

This population-based study included women with DCIS diagnosed between 2000 and 2022 from the breast cancer foundation national register (BCFNR). The register started data collection from Auckland and Waikato in 2000, expanded to Christchurch in 2009 and to Wellington in 2010, covering 63% of New Zealand’s breast cancer cases [15]. From 2020 onwards, the register expanded to include all breast cancer diagnoses nationwide. The Pharmaceutical collection (PHARMS) database contains dispensing information and medication identifiers from pharmacists for subsidised prescriptions, exclusively from medical prescriptions [18]. The PHARMS database was linked to the BCFNR through encrypted National Health Index numbers. Any patients who had a record of having invasive breast cancer before their diagnosis of DCIS were excluded (Supplementary Fig. 1).

### Variable selection and measures

Information on demographics (age at diagnosis, ethnicity, domicile code, region of surgery), clinicopathological characteristics (year of diagnosis, mode of detection, laterality, DCIS grade, DCIS size, necrosis status, circumferential surgical margin, ER testing result), and treatments (types of final surgery, RT, surgical treatment facility, ET) was extracted. Patients with more than one recorded ethnicity were allocated to a single ethnic group in order of priority: Māori, Pacific, Asian and European [19]. The deprivation level was determined based on the domicile code, using the New Zealand deprivation (NZDep) index, with 1 representing the least deprived areas and 10 representing the most deprived areas [20]. The deprivation score was grouped into NZDep 1–4, 5–7 and 8–10, and using the relevant NZDep version for the year of diagnosis: 2001 for 2000–2002, 2006 for 2004–2009, 2013 for 2010–2015, and 2018 for 2016–2022. The area of residence was categorised as rural, urban, other or unknown, according to Statistics NZ definitions based on the domicile code [21]. The region of surgery was classified as Waikato (Midland) and all other regions (Central, Northern, Southern, and Midland excluding Waikato), because clinical centres in Waikato had participated in trials evaluating ET for DCIS.

ER status and initiation of ET were grouped as ER+ with ET, ER+ without ET, ER-, and no ER testing. Time to any breast cancer event was defined as the interval from the date of DCIS diagnosis to the earliest occurrence of any of the following events: DCIS or invasive breast cancer (including locoregional recurrence and metastatic disease), death, or last follow-up for those without any breast cancer events.

### Statistical analysis

Patient demographic and clinical characteristics were presented by the receipt of ER testing. Continuous variables were reported as median with ranges, and categorical variables as frequencies with corresponding percentages. The proportion of patients tested for ER, those who were ER+ (among those tested), those who initiated ET (among ER+ cases), and the type of ET (among initiated ET cases) were calculated. Factors associated with ER testing were examined by multivariable logistic regression analyses. The proportions of patients undergoing ER testing and those initiating ET were analysed separately for the Waikato region and other regions. Because death precludes the occurrence of a subsequent breast cancer event (including both ipsilateral and contralateral invasive breast cancer or DCIS), cumulative incidence functions were estimated with death treated as a competing risk. This approach provides an unbiased estimate of the probability of experiencing a breast cancer event in the presence of the competing risk of death. Gray’s test was used to compare cumulative incidences of breast cancer event across ER status and ET groups. All analyses were performed using R version 4.3.1, and a two-sided p-value less than 0.05 was considered statistically significant. The study followed the Strengthening the Reporting of Observational Studies in Epidemiology (STROBE) reporting guideline [22].

## Results

### Characteristics of study population

Of the 5813 women with DCIS included in this study, the majority were diagnosed between the ages between 45 and 69 years (82.9%), identified as European (71.3%), lived in the least deprived areas (49.3%), or urban areas (86.3%), had their DCIS detected through the screening programme (60.5%), and were treated with BCS with RT (38.1%). The mastectomy rate was 35.1% (Table 1).

A total of 894 (15.4%) patients had an ER testing record, of whom 65.6% were from the Waikato region. The Northern region had approximately 22% of ER testing rate, while all remaining regions contributed less than 5% each (Supplementary Table 1). Among those tested, 729 (81.5%) were ER+. Among the ER+ cases, 512 (70.2%) were ER+/PR+; the remaining were PR- (17.0%), or not tested for PR (12.8%).

**Table 1:** Patient characteristics by oestrogen receptor testing for women with DCIS

	Overall *N* = 5813	No record of ER testing *N* = 4,919	Had record of ER testing *N* = 894	*p*-value^a^
**Age at diagnosis** Median (Min, Max)	56 (23, 92)	56 (26, 92)	58 (23, 91)	0.0026
**Age group**				0.0005
< 45	470 (8.1%)	398 (8.1%)	72 (8.1%)	
45–69	4,819 (82.9%)	4,108 (83.5%)	711 (79.5%)	
> 69	524 (9.0%)	413 (8.4%)	111 (12.4%)	
**Ethnicity**				< 0.0001
Māori	472 (8.1%)	349 (7.1%)	123 (13.8%)	
Pacific	264 (4.5%)	241 (4.9%)	23 (2.6%)	
Asian	831 (14.3%)	738 (15.0%)	93 (10.4%)	
European	4,147 (71.3%)	3,512 (71.4%)	635 (71.0%)	
Other or Unknown	99 (1.7%)	79 (1.6%)	20 (2.2%)	
**Year period**				0.0375
2000–2007	1,067 (18.4%)	880 (17.9%)	187 (20.9%)	
2008–2015	2,089 (35.9%)	1,795 (36.5%)	294 (32.9%)	
2016–2022	2,657 (45.7%)	2,244 (45.6%)	413 (46.2%)	
**Region of surgery**				< 0.0001
Waikato	721 (12.4%)	135 (2.7%)	586 (65.5%)	
Other regions	5092 (87.6%)	4,784 (97.3%)	308 (34.5%)	
**Deprivation**				< 0.0001
NZDep1-4 (Low deprivation)	2,867 (49.3%)	2,575 (52.3%)	292 (32.7%)	
NZDep5-7	1,658 (28.5%)	1,367 (27.8%)	291 (32.6%)	
NZDep8-10 (High deprivation)	1,288 (22.2%)	977 (19.9%)	311 (34.8%)	
**Rurality**				< 0.0001
Rural	794 (13.7%)	614 (12.5%)	180 (20.1%)	
Urban/other	5,019 (86.3%)	4,305 (87.5%)	714 (79.9%)	
**Detection**				< 0.0001
Programme screening	3,519 (60.5%)	2,957 (60.1%)	562 (62.9%)	
Non- programme imaging	1,228 (21.1%)	1,117 (22.7%)	111 (12.4%)	
Symptomatic	1,066 (18.3%)	845 (17.2%)	221 (24.7%)	
**DCIS grade**				0.1731
High	2,752 (47.3%)	2,340 (47.6%)	412 (46.1%)	
Intermediate	2,113 (36.3%)	1,769 (36.0%)	344 (38.5%)	
Low	895 (15.4%)	769 (15.6%)	126 (14.1%)	
Unknown	53 (0.9%)	41 (0.8%)	12 (1.3%)	
**DCIS size**				0.1195
≤ 20 mm	3,340 (57.5%)	2,848 (57.9%)	492 (55.0%)	
> 20 mm	2,473 (42.5%)	2,071 (42.1%)	402 (45.0%)	
**Locoregional treatment**				< 0.0001
BCS alone	1,561 (26.9%)	1,370 (27.9%)	191 (21.4%)	
BCS with RT	2,212 (38.1%)	1,786 (36.3%)	426 (47.7%)	
Mastectomy	2,040 (35.1%)	1,763 (35.8%)	277 (31.0%)	
**Facility of surgery**				< 0.0001
Private	1,766 (30.4%)	1,535 (31.2%)	231 (25.8%)	
Public	3,782 (65.1%)	3,131 (63.7%)	651 (72.8%)	
Unknown	265 (4.6%)	253 (5.1%)	12 (1.3%)	

Abbreviations: BCS: breast conserving surgery; ER: oestrogen receptor; RT: radiotherapy; NA: not applicable

^a^Kruskal-Wallis rank sum test; Pearson’s Chi-squared test

### Oestrogen receptor testing trends

The proportion of DCIS patients who had ER testing increased from 11.1% in 2000 to 23.3% in 2006, then decreased and remained relatively stable, reaching 13.9% in 2022 (Supplementary Fig. 2). Among those tested, the ER-positive rate increased from 60% in 2000 to 91.9% in 2006, after which it remained relatively stable, slightly declining to 78.7% in 2022 (Supplementary Fig. 2).

**Fig. 1:**
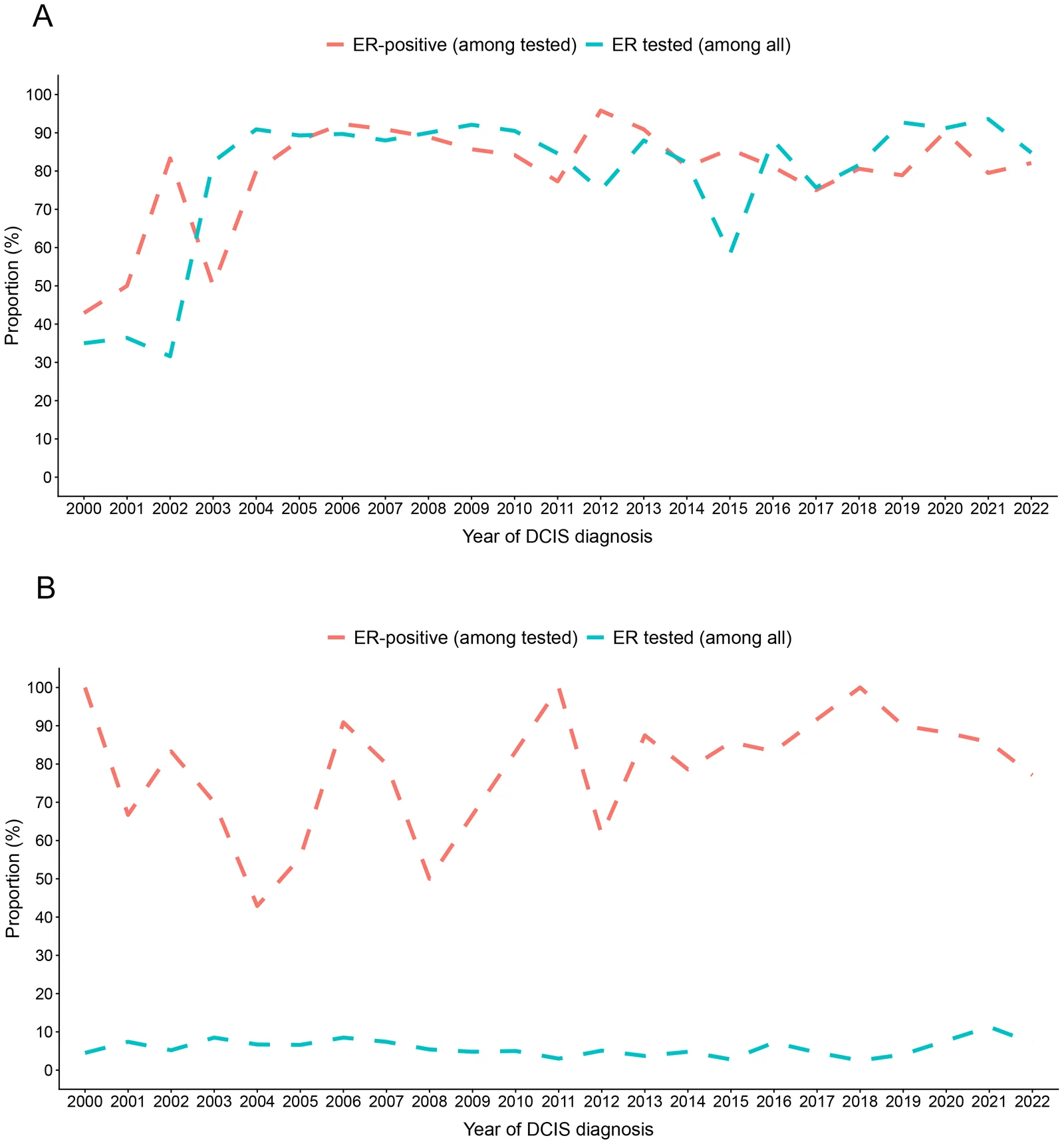
Oestrogen receptor testing status and results among tested patients, by year of diagnosis, for the Waikato region (**A**) and other regions (**B**)

In the Waikato region, ER testing increased from 35% in 1999 to 82.4% in 2003, and then stayed relatively stable, reaching 84.8% in 2022 (Fig. 1A). In other regions, ER testing rates remained low and changed little over time, increasing only from 4.5% in 2000 to 7.1% in 2022, with ER+ rates fluctuating over time ranging from 42.9% to 100% (Fig. 1B).

### Factors associated with having oestrogen receptor testing

In the Waikato region, multivariable analyses showed that those who received adjuvant RT after BCS were more likely to receive ER testing (Table 2). In contrast, women who were diagnosed in earlier calendar years or who had low DCIS grade were less likely to receive ER testing.

In other regions, ER testing was more likely among women diagnosed at an older age, Māori women, those presenting symptomatically, and those with DCIS size > 20 mm (Table 2). Conversely, women diagnosed in 2008–2015 were less likely to receive ER testing.

These associations were similar among women who had BCS, with or without RT (Supplementary Table 2).

**Table 2:** Multivariable analysis of factors associated with the odds of receiving ER testing among women with DCIS

Variables	Odds ratio for having ER testing (95%CI)	
	Waikato region *N* = 721	Other regions *N* = 5095
**Age group**		
< 45	1.35 (0.55, 3.59)	0.97 (0.62, 1.49)
45–69	1 (Reference)	1 (Reference)
> 69	1.07 (0.49, 2.42)	**2.24 (1.56**,** 3.21)**
**Ethnicity**		
Māori	1.14 (0.65, 2.11)	**1.77 (1.16**,** 2.64)**
Pacific	1.82 (0.32, 34.46)	0.89 (0.47, 1.56)
Asian	2.63 (1.00, 9.10)	1.23 (0.87, 1.71)
European	1 (Reference)	1 (Reference)
Other or unknown	0.68 (0.2, 3.2)	1.82 (0.82, 3.6)
**Year of diagnosis**		
2000–2007	**0.50 (0.30**,** 0.84)**	1.03 (0.73, 1.45)
2008–2015	0.75 (0.46, 1.22)	**0.63 (0.47**,** 0.85)**
2016–2022	1 (Reference)	1 (Reference)
**Deprivation score**		
1–4 (less deprived)	1 (Reference)	1 (Reference)
5–7	1.29 (0.76, 2.19)	1.07 (0.80, 1.42)
8–10 (more deprived)	1.15 (0.67, 1.96)	1.34 (0.97, 1.84)
**Area of residence**		
Rural	1.47 (0.88, 2.53)	1.03 (0.7, 1.48)
Urban/other	1 (Reference)	1 (Reference)
**Detection**		
Programme screening	1 (Reference)	1 (Reference)
Non- programme imaging	0.69 (0.35, 1.44)	1.01 (0.69, 1.47)
Symptomatic	0.79 (0.44, 1.44)	**2.38 (1.69**,** 3.32)**
**DCIS grade**		
High	1 (Reference)	1 (Reference)
Intermediate	0.63 (0.39, 1.01)	1.23 (0.94, 1.61)
Low	**0.47 (0.25**,** 0.88)**	1.24 (0.85, 1.80)
Unknown	0.45 (0.08, 3.53)	2.44 (0.99, 5.42)
**Tumour size**		
≤ 20 mm	1 (Reference)	1 (Reference)
>20 mm	1.23 (0.74, 2.09)	**1.84 (1.39**,** 2.44)**
**Locoregional Treatment**		
BCS alone	1 (Reference)	1 (Reference)
BCS with RT	**1.88 (1.12**,** 3.15)**	0.95 (0.68, 1.34)
Mastectomy	1.64 (0.83, 3.25)	0.88 (0.61, 1.26)
**Facility of surgery**		
Public	1 (Reference)	1 (Reference)
Private	0.73 (0.46, 1.18)	0.96 (0.73, 1.27)
Unknown	NA	1.00 (0.50, 1.84)

Abbreviation: BCS: breast conserving surgery; ER: oestrogen receptor; RT: radiotherapy; NA: not applicable

Note: Bold values denote statistical significance at the *p* < 0.05 level. N indicates the total number of patients included in each regional regression model

### Initiation of adjuvant endocrine therapy among ER+ DCIS

Among 729 patients with ER+ DCIS, 183 patients (25.1%) initiated ET; of these, 80.3% had surgery in the Waikato region (Supplementary Table 3). This regional difference in initiation rates was consistent across most demographic, tumour and treatment subgroups.

**Fig. 2:**
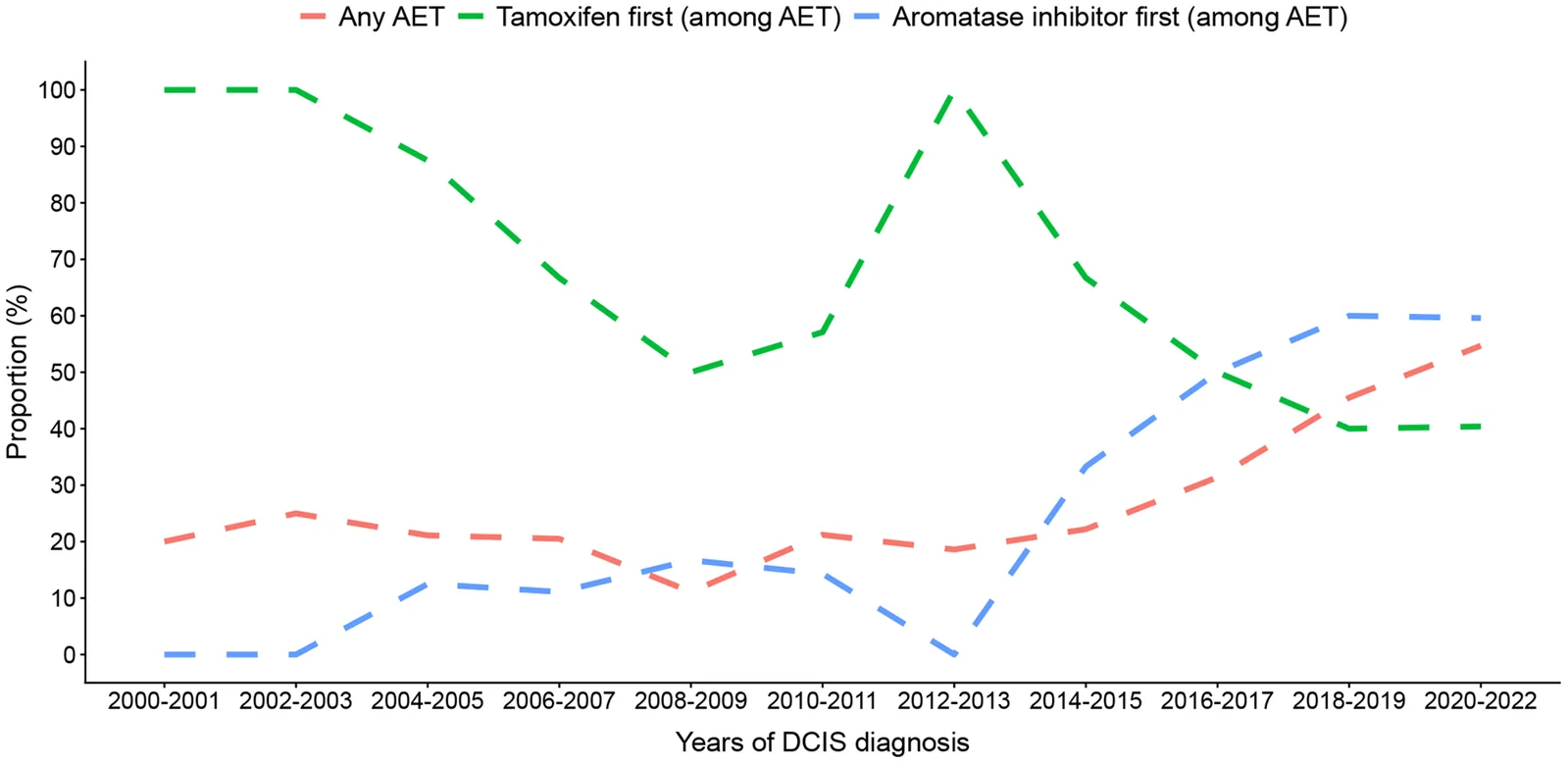
Initiation of adjuvant endocrine therapy and the type of adjuvant endocrine therapy in the Waikato region, by year of diagnosis

TAM was the most commonly used initial ET (107; 58.5%), followed by AIs (70; 38.3%); the initial ET type was unknown for six patients. In the Waikato region, the initiation of ET gradually increased from 20% in 2000–2001 to 62.5% in 2022 (Fig. 2). The initiation of AIs steadily increased from 12.5% in 2004–2005 to 55.0% in 2022.

### Breast cancer event and the use of endocrine therapy

**Fig. 3:**
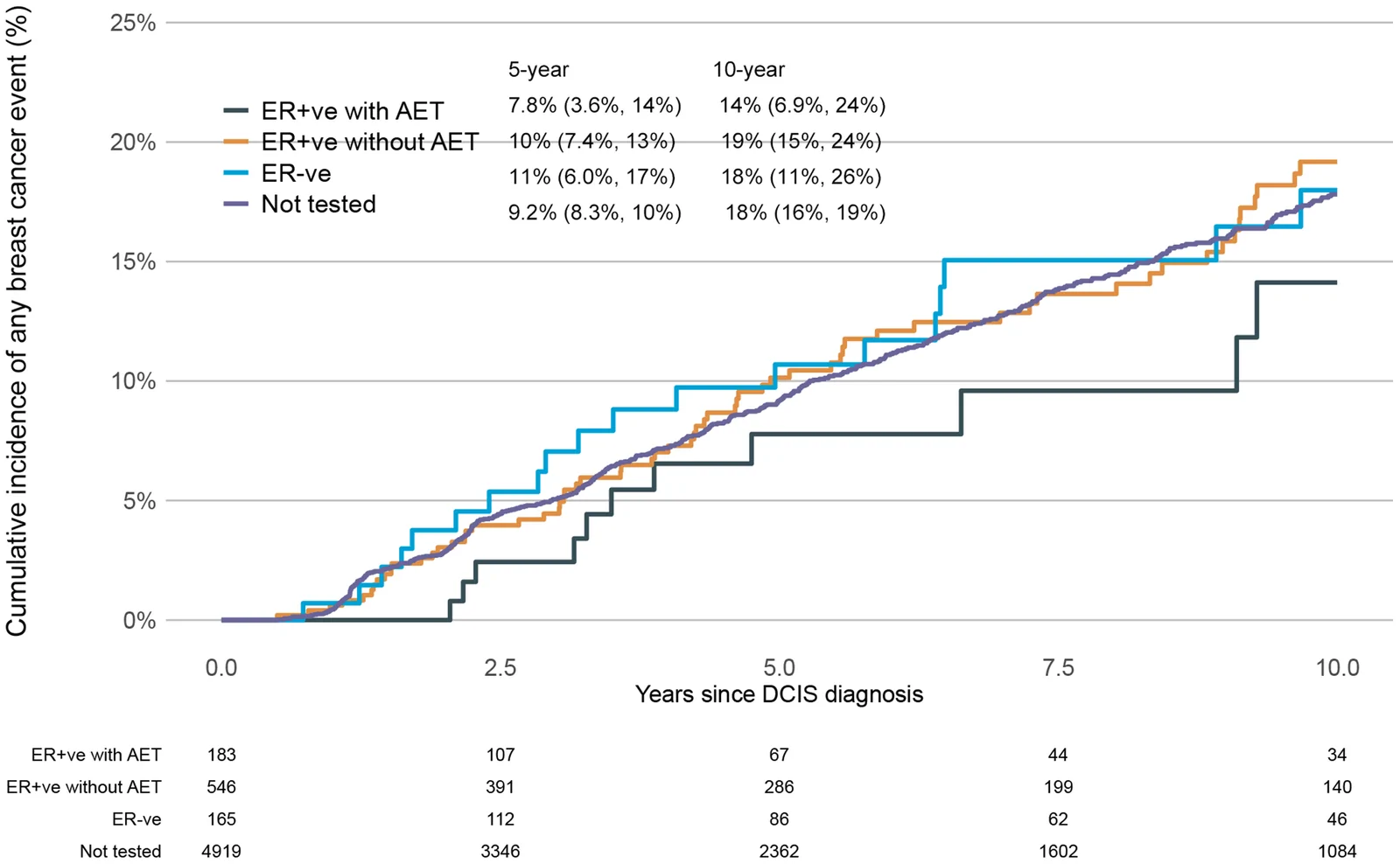
Cumulative incidence for breast cancer event by adjuvant endocrine therapy group

After a median follow-up of 4.8 years (IQR: 1.7, 9.4), no significant differences were observed in the 5-year and 10-year risk of any breast cancer event across ER status and ET groups. The cumulative risk of breast cancer events was the lowest among patients with ER+ DCIS who received ET, but there were no significant differences between groups (Fig. 3). Results were similar when the analysis was restricted to patients who had BCS, with or without RT (Supplementary Fig. 4). Among patients with ER+ DCIS and documented ET use for at least 2 years (*n* = 41), the 5-year cumulative incidence of breast cancer event was 6.3% (1.1%, 18%); however, the estimate was based on very few events and should be interpreted with caution.

## Discussion

In this population-based study, the ER testing rate for DCIS increased from 2000 to 2006, then declined before stabilising, but remained low overall (15.4%). Testing was driven predominantly by the Waikato region (81.3%), where those who received adjuvant RT after BCS were more likely to be tested. In other regions, ER testing was more likely among women diagnosed at an older age, Māori women, those presenting symptomatically, and those with DCIS size > 20 mm. Among 729 ER+ DCIS patients, 25.1% of patients initiated ET; of these, 80.3% had their diagnosis and treatment in the Waikato region.

International evidence from the US and Europe has suggested wide variations in the overall ER testing rates ranging from 30% to 85% [3, 4, 23–25]. Reported testing rates have also changed over time in response to clinical developments. In the US, ER testing increased from 17.5% in 2000–2003 to 93.1% in 2012–2014 [3], likely because TAM was approved for DCIS in 2000 based on the results from the NSABP B-24 trial [26]. The ASCO/CAP guideline initially considered ER testing for DCIS to be optional in 2010 but updated it to a recommendation in 2020, informed by the subgroup analyses of the NSABP B-24 trial showing greater benefit from TAM in ER-positive DCIS [27]. ER testing for DCIS is not standard practice in New Zealand [14]. In our cohort, the overall ER testing rate ranged from 11 to 23% during 2000–2022. Among patients who underwent ER testing, 87.2% were also tested for PR, reflecting local hormone testing practices, although PR is not recommended for DCIS by the ASCO/CAP guideline [7].

There was a regional difference in the ER testing for DCIS in New Zealand. The Waikato region had the highest testing rate of 81.3%, with a substantial increase from 35% to 83% between 2000 and 2003, whereas testing in other regions remained below 7% throughout the study period. This difference in clinical practice preferences in the Waikato region where ER testing is commonly performed for DCIS, arose from participation of local clinic centres in international clinical trials, including the UK/ANZ [28] and the IBIS-II DCIS trial [29]. The region’s established breast cancer research infrastructure, and early exposure to DCIS management with one of the two NZ breast cancer screening pilot programmes commencing in 1991, along with one of the country’s earliest breast cancer registries [30], have all supported both monitoring and adherence to best practice care. Similarly, within the non-Waikato regions, the Northern region had an ER testing rate approximately fourfold higher than that in other regions and North Shore Hospital (within the Northern region) was also a participating centre for the IBIS-II DCIS trial [29].

Among patients who underwent ER testing in the Waikato region, 82.6% were ER+ comparable to the proportion reported in the US [31]. The trend in ER positivity also mirrored the increase in ER testing over time. Fluctuations in the annual ER+ proportion in other regions over time was likely due to a small number of cases tested each year.

Since ER status is essential for determining ET eligibility, regional differences in testing patterns may reflect varying knowledge of clinical benefits, varying clinician comfort with initiation of ET and/or varying clinical expectations of treatment benefit versus adverse events. In the Waikato region, surgeons have generally managed ET for patients with DCIS, as well as for those with invasive breast cancer who do not require chemotherapy. In many other parts of NZ, and in particular Auckland, our largest population centre, ET has generally been managed by medical oncologists. Patients with DCIS may not commonly be referred to medical oncologists in NZ.

In the Waikato region, women who received BCS with RT were more likely to have ER testing. This association likely reflects the concurrent implementation of local protocols that promoted both BCS with RT and ER testing, rather than selective clinician ordering. Accordingly, these findings should be interpreted as reflecting a site-specific model of care and may not be generalisable to other New Zealand regions where different clinical pathways exist. In contrast, in other regions, where ER testing remained uncommon, testing appeared to be more selective, with a higher likelihood of testing among older women, Māori women and those presenting symptomatically or with larger DCIS lesions. These associations should be interpreted as reflecting clinician selective ordering in situations where ER status was perceived to be more likely to influence management, rather than as population-level risk factors for ER positivity or underlying disease characteristics. A previous study of invasive breast cancer has shown a higher proportion of ER+ tumour among Māori women compared to other ethnicities [32]. However, the observed higher likelihood of ER testing among Māori women outside the Waikato region should be interpreted cautiously. Given the low overall testing rate and the selective nature of ER testing in these regions, this finding may reflect local clinical practice patterns or unmeasured factors rather than a true ethnicity-related difference. Further studies with more comprehensive ER testing and clinician-level data are needed to better understand this observation. Likewise, although ER positivity in DCIS did not differ between younger and older women [33], we observed higher likelihood of ER testing among older women outside the Waikato. The reasons for this are uncertain.

Although only 25.1% patients with ER+DCIS commenced ET, TAM remained the most commonly used ET overall. However, consistent with the US study [34], we observed that, after 2018, use of AIs exceeded that of TAM. This shift in practice patterns is supported by the NSABP B-35 trial, which demonstrated that postmenopausal women with DCIS who received anastrozole for 5 years experienced fewer second breast cancer events compared to those treated with TAM [35]. However, the IBIS-II DCIS trial did not demonstrate the superiority of anastrozole over TAM [29]. Therefore, the choice of ET should consider not only efficacy but also quality of life and treatment tolerability, as AI-related musculoskeletal symptoms may negatively affect long-term adherence in some patients. Low-dose TAM (5 mg daily), which has demonstrated comparable efficacy with improved tolerability in selected populations, may also represent a reasonable option [36].We observed that patients with ER+ DCIS who received ET had the lowest cumulative risk of breast cancer event; however, the difference was not statistically significant. This may reflect limited statistical power, given the small number of treated patients and outcome events in some groups. These findings should therefore be interpreted cautiously and do not exclude a clinically meaningful benefit of ET in this cohort. Prior studies have shown that good adherence reduces recurrence in DCIS patients, including older women with a favourable prognosis [37], and that treatment for at least 2 years decreases breast events [38]. A recent US study found that, compared with ER+ DCIS who had BCS alone, BCS with ET was associated with a lower risk of subsequent invasive breast cancer events, whereas BCS with RT was not [39]. We were not able to evaluate adherence in our dataset due to the small number of patients who received ET. A previous cost-effectiveness study has found that ET may reduce healthcare costs by preventing subsequent breast cancer events in patients with ER+ DCIS, although treatment-related adverse effects may offset some of these benefits through reductions in quality-adjusted life-years [40]. ET is likely to provide the greatest value for patients who tolerate treatment well and place a high priority on reducing recurrence risk.

This regional variation in clinical practice highlights the need to update the NZ early breast cancer guideline, which was last published in 2009. In particular, the evidence regarding the efficacy of ET in DCIS from clinical trials warrants consideration when developing clearer recommendations for ER testing and the use of ET. Furthermore, a nationally consistent pathway for DCIS could build on systems already used for invasive breast cancer. Greater participation in clinical research may further strengthen the local evidence, support clinicians’ engagement with emerging evidence, and inform future guideline updates.

This is the first population-based study to investigate the trends and patterns of ER testing and initiation of ET for DCIS in New Zealand. However, interpretation of regional trends is limited by differences in the timing and completeness of registry data capture. The Waikato region was among the earliest regions to contribute data, whereas other regions began data collection later, and nationwide coverage was not achieved until 2020. Hence, ET initiation numbers in other non-Waikato regions were insufficient to present trends. Information on patients’ preferences regarding adjuvant therapy was not available. These factors may influence both clinician recommendation and patient acceptance of ET.

In conclusion, ER testing and initiation of ET for DCIS in New Zealand have slightly increased over time but remain low, except for the Waikato region, where ER testing for DCIS gradually became common practice. The observed regional variation largely reflects a single-region model of care, underscoring the need for nationally consistent guidelines and pathways rather than suggesting that the Waikato experience is directly transferable across New Zealand. The low proportion of DCIS patients with ER testing prevents clinicians and patients from making informed decisions about ET. Larger studies with longer follow-up are needed to better evaluate the effectiveness of ET in routine clinical practice. Standardizing ER testing practices and greater clinician participation in clinical trials may lead to better evidence-based care, and improve treatment options and outcomes for DCIS patients.

## Supplementary Information

Below is the link to the electronic supplementary material.

Supplementary Material 1

Supplementary Material 2

## Data Availability

The datasets used in this study contain personal information and are not publicly available, but may be requested from the Breast Cancer Foundation National Register New Zealand.
