# Supplementary material for: Oestrogen receptor testing and initiation of adjuvant endocrine therapy in women with ductal carcinoma in situ: a population-based study
Source: Breast Cancer Res Treat. 2026 Sep 2;219(2):6. doi: 10.1007/s10549-026-08072-7 (PMC13538164; doi:10.1007/s10549-026-08072-7)
Supplement: Supplementary file 1 [file 10549_2026_8072_MOESM1_ESM.docx]

**Supplementary Material**

**Oestrogen receptor testing and initiation of adjuvant endocrine therapy in women with ductal carcinoma in situ: a population-based study**

Qian Chen, Ian Campbell, Mark Elwood, Alana Cavadino, Phyu Sin Aye, Sandar Tin Tin

Supplementary Figure 1. Flow chart of patient selection

Supplementary Table 1. The oestrogen receptor testing records by region of surgery and diagnosis period.

| **Region** | **Overall** N = 894 | **2000-2007** N = 187 | **2008-2015** N = 294 | **2016-2022** N = 413 |
| --- | --- | --- | --- | --- |
| Central | 33 (3.7%) | 0 (0.0%) | 8 (2.7%) | 25 (6.1%) |
| Northern | 195 (21.8%) | 62 (33.2%) | 63 (21.4%) | 70 (16.9%) |
| Southern | 38 (4.3%) | 0 (0.0%) | 8 (2.7%) | 30 (7.3%) |
| Waikato (Midland) | 586 (65.5%) | 124 (66.3%) | 214 (72.8%) | 248 (60.0%) |
| Midland excludes Waikato | 42 (4.7%) | 1 (0.5%) | 1 (0.3%) | 40 (9.7%) |

Supplementary Figure 2. Oestrogen receptor testing status and testing result among tested patients, by year of diagnosis.

Supplementary Table 2. Multivariable analysis on factors associated with the odds of receiving ER testing among women with DCIS, who received BCS with or without RT.

| **Variables** | **Odds ratio for having ER testing (95%CI)** | |
| --- | --- | --- |
|  | **Waikato region**  **N=545** | **Other regions**  **N=3228** |
| **Age group** |  |  |
| <45 | 0.35 (0.11, 1.15) | 1.09 (0.54, 2.08) |
| 45-69 | 1 (Reference) | 1 (Reference) |
| >69 | 0.88 (0.36, 2.27) | **2.41 (1.47, 3.93)** |
| **Ethnicity** |  |  |
| Māori | 1.28 (0.67, 2.63) | **2.53 (1.54, 4.06)** |
| Pacific | NA | 0.63 (0.24, 1.4) |
| Asian | 2.25 (0.82, 8.00) | 1.2 (0.76, 1.86) |
| European | 1 (Reference) | 1 (Reference) |
| Other or unknown | 1.97 (0.33, 38.36) | 1.91 (0.64, 4.65) |
| **Year of diagnosis** |  |  |
| 2000-2007 | 0.62 (0.34, 1.13) | 0.93 (0.57, 1.48) |
| 2008-2015 | 0.74 (0.43, 1.26) | 0.61 (0.41, 0.90) |
| 2016-2022 | 1 (Reference) | 1 (Reference) |
| **Deprivation score** |  |  |
| 1-4 (less deprived) | 1 (Reference) | 1 (Reference) |
| 5-7 | 1.19 (0.64, 2.21) | 1.21 (0.83, 1.76) |
| 8-10 (more deprived) | 1.1 (0.59, 2.03) | 1.37 (0.89, 2.07) |
| **Area of residence** |  |  |
| Rural | 1.56 (0.86, 2.91) | 0.89 (0.52, 1.43) |
| Urban/other | 1 (Reference) | 1 (Reference) |
| **Detection** |  |  |
| Programme screening | 1 (Reference) | 1 (Reference) |
| Non-programme screening | 0.76 (0.34, 1.77) | 0.91 (0.55, 1.50) |
| Symptomatic | 1.09 (0.54, 2.30) | **2.45 (1.53, 3.84)** |
| **DCIS grade** |  |  |
| High | 1 (Reference) | 1 (Reference) |
| Intermediate | 0.58 (0.33, 1.01) | 1.37 (0.95, 1.99) |
| Low | **0.41 (0.20, 0.82)** | 1.25 (0.77, 2.01) |
| Unknown | 0.2 (0.02, 1.94) | 2.14 (0.66, 5.85) |
| **Tumour size** |  |  |
| ≤ 20mm | 1 (Reference) | 1 (Reference) |
| >20mm | 1.2 (0.66, 2.29) | **1.75 (1.23, 2.47)** |
| **Locoregional Treatment** |  |  |
| BCS alone | 1 (Reference) | 1 (Reference) |
| BCS with RT | **1.78 (1.04, 3.05)** | 0.97 (0.68, 1.39) |
| **Facility of surgery** |  |  |
| Public | 1 (Reference) | 1 (Reference) |
| Private | 0.72 (0.42, 1.25) | 0.89 (0.61, 1.29) |
| Unknown | NA | 1.14 (0.45, 2.54) |

Abbreviation: BCS: breast conserving surgery; RT: radiotherapy; SLNB: sentinel lymph node biopsy; NA: not applicable.

Note: Bold values denote statistical significance at the p < 0.05 level. N indicates the total number of patients included in each regional regression model.

Supplementary Table 3. Patient characteristics of initiation of AET among ER-positive DCIS, by region of surgery.

|  | **Waikato region**  **N=484** | | **Other regions**  **N=245** | |
| --- | --- | --- | --- | --- |
| **Variables** | **Without AET** N = 337 | **With AET** N = 147 | **Without AET** N = 209 | **With AET** N = 36 |
| **Age at diagnosis**  Median (Min, Max) | 58 (33, 87) | 54 (35, 85) | 60 (23, 91) | 56 (40, 79) |
| **Age group** |  |  |  |  |
| <45 | 14 (48.3%) | 15 (51.7%) | 16 (72.7%) | 6 (27.3%) |
| >69 | 23 (67.6%) | 11 (32.4%) | 51 (96.2%) | 2 (3.8%) |
| 45-69 | 300 (71.3%) | 121 (28.7%) | 142 (83.5%) | 28 (16.5%) |
| **Ethnicity** |  |  |  |  |
| Māori | 55 (71.4%) | 22 (28.6%) | 20 (74.1%) | 7 (25.9%) |
| Pacific | 5 (55.6%) | 4 (44.4%) | 10 (90.9%) | 1 (9.1%) |
| Asian | 30 (78.9%) | 8 (21.1%) | 37 (90.2%) | 4 (9.8%) |
| European | 245 (70.0%) | 105 (30.0%) | 135 (84.9%) | 24 (15.1%) |
| Other or Unknown | 2 (20.0%) | 8 (80.0%) | 7 (100.0%) | 0 |
| **Detection** |  |  |  |  |
| BSA screen | 257 (72.0%) | 100 (28.0%) | 96 (83.5%) | 19 (16.5%) |
| non-BSA screen | 25 (53.2%) | 22 (46.8%) | 38 (82.6%) | 8 (17.4%) |
| Symptomatic | 55 (68.8%) | 25 (31.3%) | 75 (89.3%) | 9 (10.7%) |
| **DCIS Grade** |  |  |  |  |
| High | 115 (58.7%) | 81 (41.3%) | 65 (77.4%) | 19 (22.6%) |
| Intermediate | 155 (73.8%) | 55 (26.2%) | 89 (85.6%) | 15 (14.4%) |
| Low | 64 (86.5%) | 10 (13.5%) | 48 (98.0%) | 1 (2.0%) |
| Unknown | 3 (75.0%) | 1 (25.0%) | 7 (87.5%) | 1 (12.5%) |
| **DCIS size** |  |  |  |  |
| ≤20mm | 228 (74.3%) | 79 (25.7%) | 104 (89.7%) | 12 (10.3%) |
| >20mm | 109 (61.6%) | 68 (38.4%) | 105 (81.4%) | 24 (18.6%) |
| **Locoregional treatment** |  |  |  |  |
| BCS alone | 77 (78.6%) | 21 (21.4%) | 72 (93.5%) | 5 (6.5%) |
| BCS with RT | 184 (66.2%) | 94 (33.8%) | 59 (80.8%) | 14 (19.2%) |
| Mastectomy | 76 (70.4%) | 32 (29.6%) | 78 (82.1%) | 17 (17.9%) |
| **Facility of surgery** |  |  |  |  |
| Private | 68 (56.7%) | 52 (43.3%) | 51 (79.7%) | 13 (20.3%) |
| Public | 269 (73.9%) | 95 (26.1%) | 149 (87.6%) | 21 (12.4%) |
| Unknown | 0 | 0 | 9 (81.8%) | 2 (18.2%) |

Supplementary Figure 3. The initiation of adjuvant endocrine therapy and the type of adjuvant endocrine therapy in all regions, by year of diagnosis.

Supplementary Figure 4. Cumulative incidence for breast cancer event by adjuvant endocrine therapy group, among patients who had BCS, with or without RT.
